# Supplementary material for: A Transect Through the Living Environments of Slovakia’s Roma Population: Urban, Sub-Urban, and Rural Settlements, and Exposure to Environmental and Water-Related Health Risks
Source: Int J Environ Res Public Health. 2025 Jun 23;22(7):988. doi: 10.3390/ijerph22070988 (PMC12294940; doi:10.3390/ijerph22070988)
Supplement: Supplementary file 1 [file ijerph-22-00988-s001.zip › ijerph-3667584-supplementary.pdf]

Supplementary Materials

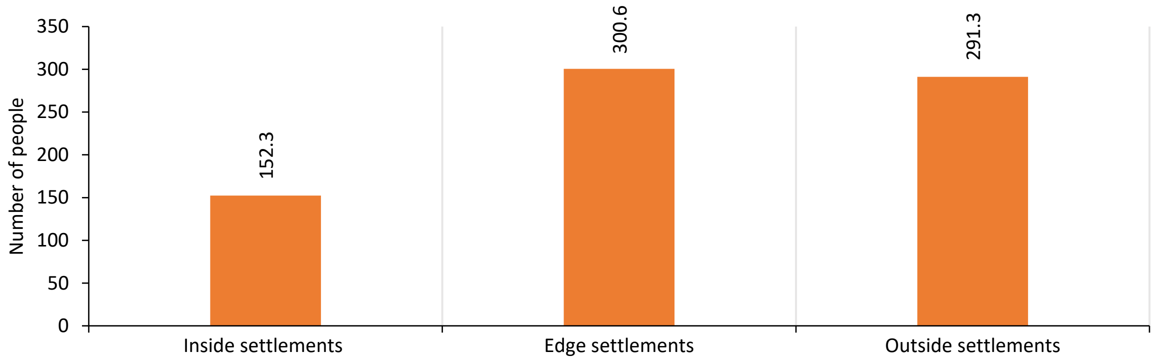

Figure S1. The average number of inhabitants living in settlements grouped according to the localization of the settlement in the municipality.

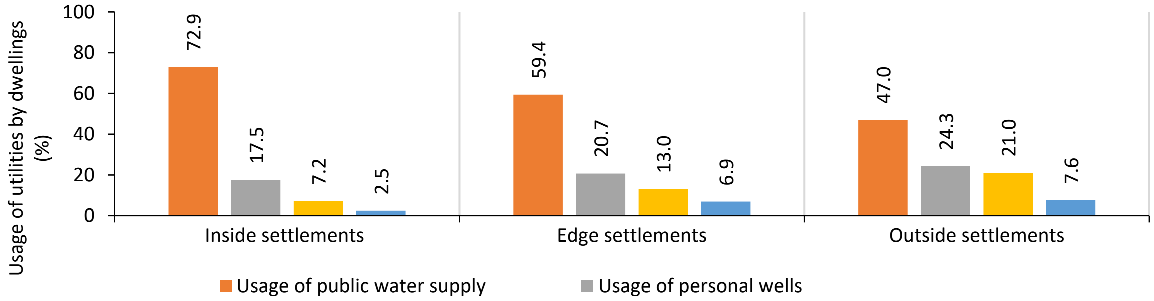

Figure S2. The average value of the usage of different water sources in dwellings in settlements grouped according to the localization of the settlement in the municipality.

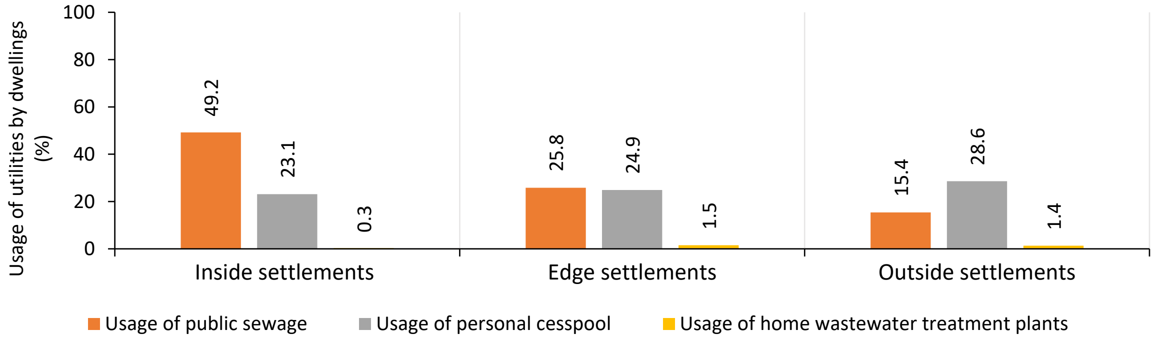

Figure S3. The average value of the usage of biological waste management in dwellings in settlements grouped according to the localization of the settlement in the municipality.

Table S1. Energy situation in different Roma living environments at different levels of centrality.

Natural Disasters						
Occurrence of Disaster	Frequencies of Settlements (%)					
	Inside Settlements		*Edge* Settlements		*Outside* Settlements	
	Flooding	Other	Flooding	Other	Flooding	Other
Yes	16.01	8.99	32.67	23.11	25.26	15.98
No	83.99	91.01	67.33	76.89	74.74	84.02

Table S2. Distance to healthcare in different Roma living environments at different levels of centrality.

Medical Facilities						
Distance (km)	Frequencies of Settlements					
	Inside Settlements		*Edge* Settlements		*Outside* Settlements	
	GP Office	Paediatrician	GP Office	Paediatrician	GP Office	Paediatrician
0–1	70.22	57.58	40.44	32.67	21.65	17.53
1–3	8.71	8.71	15.34	12.95	22.16	17.01
3–7	12.92	14.33	26.29	25.10	35.05	32.99
7–15	8.15	14.33	16.14	23.31	18.04	24.74
>15	0.00	5.06	1.79	5.98	3.09	7.73

Table S3. Frequency of settlements as a percentage value in different levels of centrality with different distance from the public transport.

Distance From the Public Transport						
Distance (km)	Frequencies of Settlements (%)					
	Inside Settlement		*Edge* Settlements		*Outside* Settlements	
	Bus	Train	Bus	Train	Bus	Train
0–1	98.31	48.88	92.03	34.66	86.60	31.44
1–3	1.69	11.52	3.98	14.34	10.31	11.86
3–7	0.00	13.76	3.78	11.95	2.58	25.77
7–15	0.00	15.73	0.20	20.72	0.52	13.92
>15	0.00	10.11	0.00	18.33	0.00	17.01

Table S4. Housing situation in different Roma living environments at different levels of centrality.

Number of Unapproved Houses									
Number of Houses	Frequencies of Settlements (%)								
	Inside Settlements			*Edge* Settlements			*Outside* Settlements		
	Brick	Wooden	Huts	Brick	Wooden	Huts	Brick	Wooden	Huts
0–5	92.13	99.44	94.66	75.70	95.62	82.27	74.74	95.88	78.87
5–10	3.09	0.28	3.09	8.76	1.59	5.98	8.25	0.52	5.15
10–15	1.97	0.00	0.84	2.79	1.00	1.39	4.12	1.03	1.03
15–20	1.12	0.00	0.56	1.99	0.20	1.99	2.58	0.00	2.58
20–30	0.56	0.00	0.56	3.98	0.80	2.79	4.64	0.52	5.15
>30	1.12	0.28	0.28	6.77	0.80	5.58	5.67	2.06	7.22

Table S5. Frequency of settlements as a percentage value in different levels of centrality with a percentage value of number of dwellings that uses different utilities such as public electricity and gas line.

Usage of Utilities						
Dwellings That Use Utilities (%)	Frequencies of Settlements (%)					
	Inside Settlements		*Edge* Settlements		*Outside* Settlements	
	Electricity	Gas	Electricity	Gas	Electricity	Gas
0–20	2.81	62.92	7.97	78.29	11.34	93.81
20–40	1.12	4.49	2.19	3.19	2.58	3.09
40–60	3.93	5.90	8.17	6.37	6.70	0.52
60–80	6.74	7.58	7.57	5.38	10.31	1.03

80–100	85.39	19.10	74.10	6.77	69.07	1.55

Table S6. Prevalence of endoparasites in human stool samples from selected localities in Slovakia.

	**Overall**	**Majority**	***Inside* Settlements/Village**	***Edge* Settlements**	***Outside* Settlements**
Collected samples	5378	2896	643	1244	595
Positive samples	476	17	131	178	150
Negative samples	4902	2879	512	1066	445
% positivity	8.85	0.59	20.37	14.31	25.21
95% CI	8.89–10.57	0.34–0.93	17.32–23.69	12.40–16.37	21.76–28.90
OR	-	0.02 [A]	1.17 [B]	0.56 [C]	1.72 [D]
95% CI	-	0.01-0.04	0.94-1.47	0.46-0.69	1.37-2.14
χ^2	-	-	2.035109	28.97192	23.42523
p-value (χ^2)	-	-	0.1537031	7.34E-08 *	1.30E-06 *

95% CI: 95% confidence interval; OR: odds ratio; * statistically significant (P < 0.001); [A]: odds ratio calculated between Non-Roma group and sum of the settlements localization as a refence group; [B]: odds ratio calculated between *Inside* settlements/village settlements and sum of Non-Roma with Edge and *Outside* settlements as a refence group; [C]: odds ration calculated between *Edge* settlements and sum of Non-Roma with *Inside* settlements/village and *Outside* settlements as a refence group; D: odds ratio calculated between *Outside* settlements and sum of Non-Roma with *Inside* settlements/village and *Edge* settlements as a refence group.

Table S7. Prevalence of soil-transmitted helminths in dog faecal samples and soil samples from selected localities in Slovakia.

	**Dogs Samples**	**Soil Samples**	**Dogs Samples**	**Soil Samples**	**Dogs Samples**	**Soil Samples**	**Dogs Samples**	**Soil Samples**
	**Overall**		***Inside* Settlements/Village**		***Edge* Settlements**		***Outside* Settlements**	
Collected samples	2764	710	2076	398	272	190	416	122
Positive samples	1153	250	762	46	134	134	257	70
Negative samples	1611	460	1314	352	138	56	159	52
% positivity	41.71	35.21	36.71	11.56	49.26	70.53	61.78	57.38
95% CI	39.86–43.57	31.69–38.85	34.62–38.82	8.58–15.11	43.17–55.36	63.49–76.90	56.91-66.47	48.10–66.28
OR	-	-	0.44 [A]	0.12 [A]	1.4 [B]	8.33 [B]	2.61 [C]	3.05 [C]
95% CI	-	-	0.36–0.52	0.08–0.175	1.09-1.80	5.73-12.11	2.11-3.24	2.04-4.54
χ^2	-	-	86.08991	148.2602	7.072631	141.8231	81.0806	31.72677
p-value (χ^2)	-	-	1.72E-20 *	4.16E-34 *	0.007827022	1.06E-32 *	2.17E-19 *	1.77E-08 *

95% CI: 95% confidence interval; OR: odds ratio; * statistically significant (P < 0.001); [A]: odds ratio calculated between *Inside* settlements/village group and sum of the Edge and *Outside* settlements localization as a refence group; [B]: odds ratio calculated between *Edge* settlements and sum of *Inside* settlements/village group and *Outside* settlements as a refence group; [C]: odds ratio calculated between *Outside* settlements and sum of *Inside* settlements/village group and *Edge* settlements as a refence group.
